# Biotransformation of the high-molecular weight polycyclic aromatic hydrocarbon (PAH) benzo[*k*]fluoranthene by Sphingobium sp. strain KK22 and identification of new products of non-alternant PAH biodegradation by liquid chromatography electrospray ionization tandem mass spectrometry

**DOI:** 10.1111/1751-7915.12102

**Published:** 2013-12-11

**Authors:** Allyn H Maeda, Shinro Nishi, Yuji Hatada, Yasuhiro Ozeki, Robert A Kanaly

**Affiliations:** 1 Department of Life and Environmental System Science, Graduate School of Nanobiosciences, Yokohama City University22-2 Seto, Kanazawa, Yokohama, 236-0027, Japan; 2 Institute of Biogeosciences (Biogeos), Japan Agency for Marine-Earth Science and Technology (JAMSTEC)2-15 Natsushima-cho, Yokosuka, 237-0061, Japan

## Abstract

A pathway for the biotransformation of the environmental pollutant and high-molecular weight polycyclic aromatic hydrocarbon (PAH) benzo[*k*]fluoranthene by a soil bacterium was constructed through analyses of results from liquid chromatography negative electrospray ionization tandem mass spectrometry (LC/ESI(–)-MS/MS). Exposure of *Sphingobium* sp. strain KK22 to benzo[*k*]fluoranthene resulted in transformation to four-, three-and two-aromatic ring products. The structurally similar four-and three-ring non-alternant PAHs fluoranthene and acenaphthylene were also biotransformed by strain KK22, and LC/ESI(–)-MS/MS analyses of these products confirmed the lower biotransformation pathway proposed for benzo[*k*]fluoranthene. In all, seven products from benzo[*k*]fluoranthene and seven products from fluoranthene were revealed and included previously unreported products from both PAHs. Benzo[*k*]fluoranthene biotransformation proceeded through *ortho*-cleavage of 8,9-dihydroxy-benzo[*k*]fluoranthene to 8-carboxyfluoranthenyl-9-propenic acid and 9-hydroxy-fluoranthene-8-carboxylic acid, and was followed by *meta*-cleavage to produce 3-(2-formylacenaphthylen-1-yl)-2-hydroxy-prop-2-enoic acid. The fluoranthene pathway converged with the benzo[*k*]fluoranthene pathway through detection of the three-ring product, 2-formylacenaphthylene-1-carboxylic acid. Production of key downstream metabolites, 1,8-naphthalic anhydride and 1-naphthoic acid from benzo[*k*]fluoranthene, fluoranthene and acenaphthylene biotransformations provided evidence for a common pathway by strain KK22 for all three PAHs through acenaphthoquinone. Quantitative analysis of benzo[*k*]fluoranthene biotransformation by strain KK22 confirmed biodegradation. This is the first pathway proposed for the biotransformation of benzo[*k*]fluoranthene by a bacterium.

## Introduction

Benzo[*k*]fluoranthene is a genotoxic, five-ring, non-alternant high-molecular weight polycyclic aromatic hydrocarbon (HMW PAH) and environmental pollutant. It is commonly found at petrochemical processing, creosote production and coal gasification sites; however, it is also globally distributed and may originate from non-anthropogenic sources (Georgiadis *et al*., 2000; Boström *et al*., 2002; Baraniecka *et al*., 2010; Lladó *et al*., 2013). In animals, there is sufficient evidence for carcinogenicity caused by benzo[*k*]fluoranthene, and in humans it is classified as a group 2B carcinogen by the International Agency for Research on Cancer (Boström *et al*., 2002). At the same time, it is classified as a probable human carcinogen according to the U.S. Agency for Toxic Substances and Disease Registry (ATSDR, 1995). Benzo[*k*]fluoranthene inductively enhances cytochrome P4501A1 and 1A2 levels (Vakharia *et al*., 2001a,b) and has been shown to be mutagenic, tumorigenic and forms DNA adducts (LaVoie *et al*., 1982; Weyand *et al*., 1987; 1988; Ericson *et al*., 1999).

Structurally, benzo[*k*]fluoranthene may be viewed as a single-or double-benzannelated derivative of the four-ring and three-ring PAHs fluoranthene and acenaphthylene respectively. These three molecules constitute a series of non-alternant PAHs whereby the combination of five-and six-membered rings that comprise their structures is also the structural motif upon which many new materials that are under development are based, including organic light-emitting diodes, fullerenes and in the termination of single-wall carbon nanotubes (Birer *et al*., 2007; Xia *et al*., 2010; Saranya *et al*., 2011). As a member of the five-ring HMW PAH class, benzo[*k*]fluoranthene is considered to be more resistant to biotransformation partly due to its molecular stability, hydrophobicity and low water solubility, which is approximately 1 ug L^−1^ or less (Cerniglia, 1992; De Maagd *et al*., 1998; Sverdrup *et al*., 2002). In the environment, it occurs in polluted soils in the parts per million range, displays varying levels of environmental persistence and is included as one of 16 pollutants on the U.S. Environmental Protection Agency's (EPA) Priority Pollutant List (Van Brummelen *et al*., 1996; Hawthorne and Grabanski, 2000; Sasek *et al*., 2003; Potin *et al*., 2004; Li *et al*., 2008; Wang *et al*., 2010; Achten *et al*., 2011; Lladó *et al*., 2013). Considering all of these factors, there is much interest to understand the pathways by which benzo[*k*]fluoranthene may be transformed in the environment and to predict the potential mobility of its biotransformation products. One of the first steps to develop these understandings is to determine the nature of benzo[*k*]fluoranthene biotransformation products that may be produced by soil microorganisms.

There are few reports of the biodegradation of PAHs with five or more rings by isolated bacterial strains and even fewer reports of their biotransformation products (Kanaly and Harayama, 2000; 2010). To the best of our knowledge, a pathway that documents the prokaryotic biotransformation of benzo[*k*]fluoranthene has not been reported. A single metabolite from benzo[*k*]fluoranthene biodegradation, 9-hydroxy-fluoranthene-8-carboxylic acid, was proposed in 1997 after gas chromatography–mass spectrometry (GC-MS) analyses of extracts from a mixed bacterial culture that was growing on a nutrient solution in an oil-in-water system with dodecane (Preuß *et al*., 1997).

In contrast, pathways that describe the bacterial biotransformation of the structurally analogous four-ring and three-ring non-alternant PAHs fluoranthene and acenaphthylene have been proposed in previous studies. In the case of fluoranthene, many biotransformation products have been identified that have originated from gram-positive *Mycobacteria* (Kelley *et al*., 1991; 1993; Rehmann *et al*., 2001; López *et al*., 2005; 2006; Kweon *et al*., 2007). In regard to the gram-negative sphingomonads, which are also known for their PAH-degrading capabilities, there are fewer fluoranthene biotransformation products reported, and these have been documented from only four strains and all from the genus *Sphingomonas*. In the most complete pathway proposed, *Sphingomonas paucimobilis* sp. strain EPA505 was reported to biotransform fluoranthene to six metabolites via initial 7,8-carbon position dioxygenation to form 7,8-dihydroxyfluoranthene, which was followed by *meta*-ring cleavage to 2-hydroxyacenaphthylene-1-carboxylic acid, 1-acenaphthylene-1(2*H*)-one, acenaphthoquinone, naphthalene-1,8-dicarboxylic acid (detected as 1,8-naphthalic anhydride) and 2,3-dihydroxy-benzoic acid (Ho *et al*., 2000; Story *et al*., 2001). Documentation of fluoranthene biotransformation products that originated from 7,8-and 1,2-carbon position attacks on the parent molecule were also reported for *Sphingomonas* sp. strain LB126 (van Herwijnen *et al*., 2003; Schuler *et al*., 2008), *Sphingomonas* sp. strain VKM B-2434 (Baboshin *et al*., 2008) and *Sphingomonas* sp. strain PheB4 (Zhong *et al*., 2007). Finally, fluoranthene biotransformation through cloned/expressed proteins of *Sphingomonas* sp. strain CHY-1 were also documented (Jouanneau and Meyer, 2006; Jouanneau *et al*., 2006). Acenaphthylene, the three-ring analogue of benzo[*k*]fluoranthene, was biotransformed to acenaphthoquinone via *cis*-1,2-acenapthenediol and 1,2-dihydroxyacenaphthylene by *Sphingobium yanoikuyae* strain B1; however, ring cleavage products were not detected (Schocken and Gibson, 1984).

Liquid chromatography coupled with electrospray ionization tandem mass spectrometry (LC/ESI-MS/MS) has become a useful tool in a variety of fields including in the pharmaceutical industry for metabolite identification. Although it has not been applied to a large degree in the field of environmental microbiology, it has high potential because mass and structural information of biotransformation products may be obtained through controlled fragmentations. At the same time, ESI is amenable for the analysis of polar compounds, and sample derivatization is not necessary. In this report, LC/ESI-MS(/MS) was utilized to investigate the biotransformation of the HMW PAH benzo[*k*]fluoranthene by the soil bacterial isolate *Sphingobium* species strain KK22. Biotransformations of the structurally analogous PAHs fluoranthene and acenaphthylene were also investigated, and all results were corroborated to propose a pathway for the biotransformation of benzo[*k*]fluoranthene by a bacterium for the first time.

## Results

### Detection of HMW tetraaromatic acid products of benzo[*k*]fluoranthene

**Figure 1:**
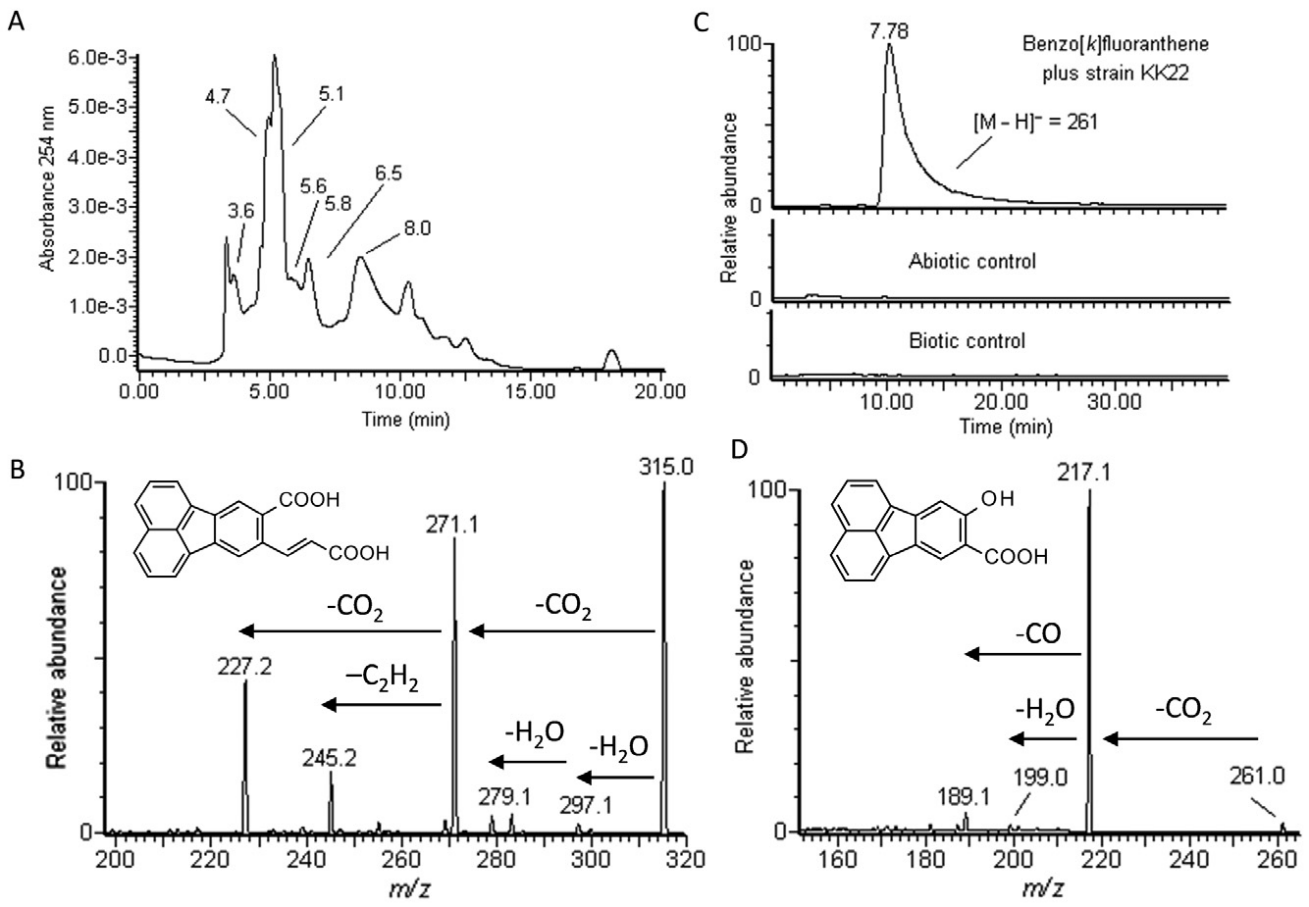
Results of LC/ESI(–)-MS(/MS) analyses of extracts from strain KK22 exposure to benzo[*k*]fluoranthene.A. Absorbance at 254 nm.B. Fragmentation pattern acquired from product ion scan analysis of the deprotonated molecule [M – H]^−^ = 315.C. Extracted ion chromatograms from full scan analyses that compare the relative abundances of the biotransformation product that corresponded to the deprotonated molecule [M – H]^−^ = 261 as detected in exposed cells and abiotic and biotic controls.D. Fragmentation pattern acquired from product ion scan analysis of the deprotonated molecule [M – H]^−^ = 261 of exposed cells from C above. Molecular structures proposed for each product are shown. Details are given in Table 1.

Results of liquid chromatography with ultraviolet detection (LC-UV) analysis of *t* = 10 day extracts from strain KK22 cells exposed to benzo[*k*]fluoranthene are shown in Fig. 1A whereby multiple peaks over a retention time (*t_R_*) range from approximately 3 to 20 min were revealed. Peaks in Fig. 1A are labeled by their respective *t_R_* and represent biotransformation products examined in this study. The biotransformation product with the greatest molar mass, corresponding to the deprotonated molecule, [M – H]^−^ = 315, eluted after 5.8 min. Product ion scan analysis of this biotransformation product revealed two abundant fragmentation ions at *m/z* 271 and *m/z* 227, which corresponded to losses of 44 and 88 Da each from the parent deprotonated molecule, respectively, and represented sequential losses of CO_2_ (Fig. 1B). A strong product ion was also revealed at *m/z* 245, which indicated sequential losses of 44 and 26 Da from the parent deprotonated molecule and represented losses of CO_2_ and C_2_H_2,_ and provided further evidence for the structure of a tetraaromatic *ortho*-ring cleavage product as proposed in Fig. 1B. Based upon a molecular formula of C_20_H_12_O_4_, it appeared that the biotransformation product that corresponded to [M – H]^−^ = 315 was 8-carboxyfluoranthenyl-9-propenic acid, an *ortho*-ring cleavage product of 8,9-dihydroxy-benzo[*k*]fluoranthene.

**Table 1:** Fragmentation ions revealed by LC/ESI(–)-MS/MS product ion scan analyses of metabolites produced from the biotransformation of benzo[*k*]fluoranthene by strain KK22

Parent ion [M – H]^−^	*t_R_*^a^ (min)	CID^b^ (eV)	Diagnostic fragments from product ion scan analyses (% relative intensity)	Identity assignment
315	5.8	8	315 (M^−^, 100), 297 (M^−^ – H_2_O, 2), 283 (5), 279 (M^−^ – 2H_2_O, 5), 271 (M^−^ – CO_2_, 84) 245 (M^−^ – CO_2_ – C_2_H_2_, 17), 227 (M^−^ – 2CO_2_, 43)	8-Carboxyfluoranthenyl-9-propenic acid
265	5.1	20	265 (M^−^, 61), 221 (M^−^ – CO_2_, 11), 193 (M^−^ – CO_2_ – CO, 50), 175 (M^−^ – CO_2_ – CO – H_2_O, 17), 165 (M^−^ – CO_2_ – 2CO, 3), 97 (HC_4_O_3_^−^, 100)	3-(2-Formylacenaphthylen-1-yl)-2-hydroxy-prop-2-enoic acid
261	8.0	20	261 (M^−^, 3), 217 (M^−^ – CO_2_, 100), 199 (M^−^ – CO_2_ – H_2_O, 0.1), 189 (M^−^ – CO_2_ – CO, 1), 173 (1)	9-Hydroxy-fluoranthene-8-carboxylic acid
239	3.6	8	239 (M^−^, 100), 195 (M^−^ – CO_2_, 95), 151 (M^−^ – 2CO_2_, 53)	Acenaphthylene-1,2-dicarboxylic acid
229	5.6	8	229 (M^−^, 100), 215 (2), 197 (M^−^ – CH_3_OH, 15)^c^, 185 (M^−^ – CO_2_, 13), 171 (5), 157 (M^−^ – CO_2_ – CO, 77), 127 (6)	1,8-Naphthalic anhydride^d^
223	4.7	20	223 (M^−^, 4), 179 (M^−^ – CO_2_, 100), 151 (M^−^ – CO, 91)	2-Formylacenaphthylene-1-carboxylic acid^e^
171	6.5	8	171 (M^−^, 100), 153 (M^−^ – H_2_O, 7), 127 (M^−^ – CO_2_, 8)	1-Naphthoic acid^d^

a *t_R_*, retention time.

b Collision-induced dissociation energy.

c The product corresponding to [M – H]^−^ = 229 was detected as its methanol adduct.

d Products were detected from benzo[*k*]fluoranthene, fluoranthene and acenaphthylene biotransformation.

e Product was detected from benzo[*k*]fluoranthene and fluoranthene biotransformation.

A second tetraaromatic acid compound was revealed to occur in relatively high abundance in cultures where strain KK22 was exposed to benzo[*k*]fluoranthene compared with the abiotic and biotic controls. It corresponded to the deprotonated molecule [M – H]^−^ = 261, *t_R_* = 7.8–8.0 min, and the normalized extracted ion chromatograms for this deprotonated molecule from each of the three culture conditions at T = 10 days is given in Fig. 1C for comparison. Product ion scan analysis of [M – H]^−^ = 261 revealed a strong fragment ion at *m/z* 217 (loss of 44 Da) and weaker ions at *m/z* 189 (loss of 72 Da) and *m/z* 199 (loss of 62 Da), which were indicative of losses of CO_2_, CO_2_ plus CO and CO_2_ plus H_2_O respectively. *o*-Hydroxy-triaromatic acids and *o*-hydroxy-naphthoic acids such as those produced during bacterial benz[*a*]anthracene biodegradation are known to result in losses of CO_2_, CO and H_2_O from the parent molecule upon mass fragmentation (Mahaffey *et al*., 1988; Kunihiro *et al*., 2013). At the same time, application of ESI negative ionization and collision-induced dissociation (CID) MS/MS to fragment hydroxylated PAHs revealed that losses of a carbonyl group as 28 Da were a common occurrence (Xu *et al*., 2004). Based upon these considerations, it was concluded that the biotransformation product that corresponded to [M – H]^−^ = 261 was 9-hydroxyfluoranthene-8-carboxylic acid, C_17_H_10_O_3_. In the only study to document a bacterial biotransformation product of benzo[*k*]fluoranthene, 9-hydroxyfluoranthene-8-carboxylic acid was detected as its tetramethylsilane derivative by GC-MS from a mixed culture (Preuß *et al*., 1997).

### Detection of three-and two-ring products of benzo[*k*]fluoranthene

**Figure 2:**
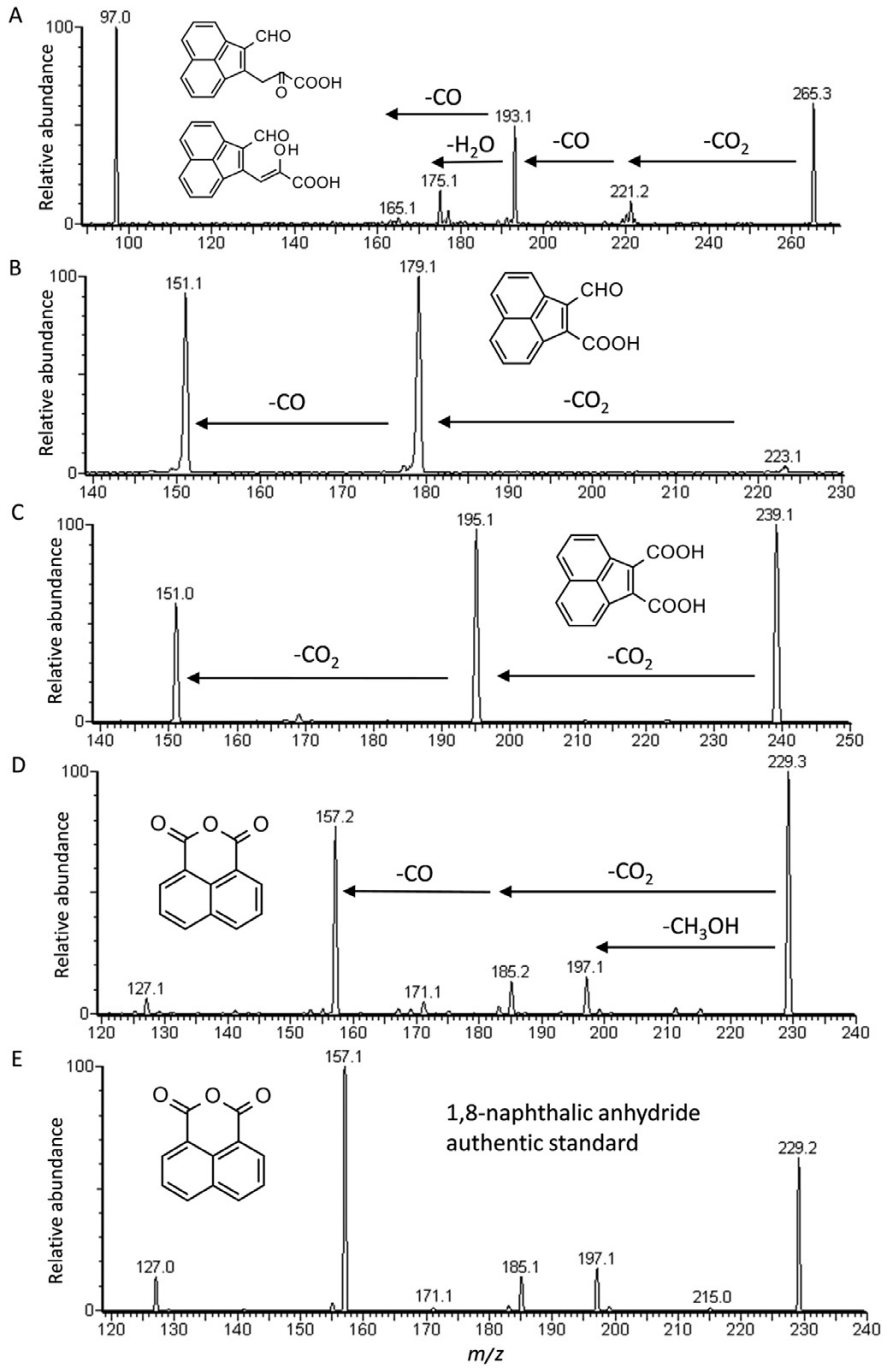
Results of LC/ESI(–)-MS/MS product ion scan analyses of extracts from strain KK22 exposure to benzo[*k*]fluoranthene. Fragmentation patterns acquired from the deprotonated molecules: (A) [M – H]^−^ = 265; (B) [M – H]^−^ = 223; (C) [M – H]^−^ = 239; (D) [M – H]^−^ = 229; and (E) an authentic standard of 1,8-naphthalic anhydride, [M – H]^−^ = 229. Molecular structures proposed for each product are shown. Details for all acquisitions are given in the text and Table 1.

**Figure 3:**
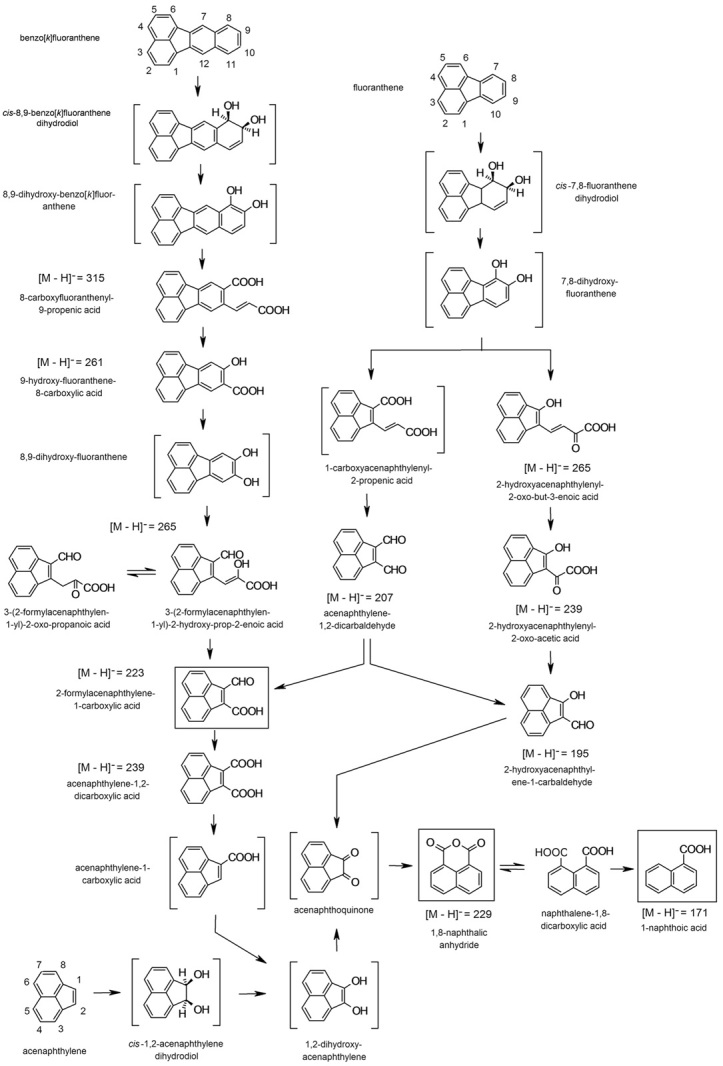
Pathways proposed for the biotransformation of the HMW PAHs benzo[*k*]fluoranthene and fluoranthene and the PAH acenaphthylene by *Sphingobium* sp. strain KK22. Products in brackets were not identified in the culture medium. Products in boxes were detected in extracts from benzo[*k*]fluoranthene and fluoranthene biodegradation, [M – H]^−^ = 223, or in extracts from benzo[*k*]fluoranthene, fluoranthene and acenaphthylene biodegradation, [M – H]^−^ = 229 and [M – H]^−^ = 171.

In addition to [M – H]^−^ = 315 and [M – H]^−^ = 261, full scan mass screening analyses resulted in five other putative target ions of interest with values ranging from [M – H]^−^ = 265 to [M – H]^−^ = 171 (Table 1). Shown in Fig. 2A are the results of product ion scan analysis of the benzo[*k*]fluoranthene product that corresponded to [M – H]^−^ = 265, *t_R_* = 5.1, where losses of CO_2_ (*m/z* 221), CO_2_ and CO (*m/z* 193), CO_2_ and 2CO (*m/z* 165), and CO_2_, CO and H_2_O (*m/z* 175) were revealed. Fragment ion *m/z* 97 occurred in greatest abundance and appeared to be the result of loss of the alpha-keto acid side chain and aryl carbon from the proposed molecular structure shown in Fig. 2A. Further support for this assignment is given by the lack of detection of fragmentation ion *m/z* 151 that corresponded to the acenaphthylene fragmentation ion as discussed in the following section. Overall, considering that this fragmentation pattern included sequential losses of 44 Da (CO_2_) and 56 Da (2CO) and a molecular formula of C_16_H_10_O_4_, a *meta*-ring cleavage product derived from an upstream dihydroxy-fluoranthene metabolite such as 8,9-dihydroxy-fluoranthene was proposed. These structures (Fig. 2A) are in agreement with the structures of the proposed upstream biotransformation products [M – H]^−^ = 315 and [M – H]^−^ = 261, which occurred because of initial oxidative attack of the benzo[*k*]fluoranthene molecule at the 8,9-carbon positions to produce *cis*-8,9-benzo[*k*]fluoranthene-dihydrodiol and 8,9-dihydroxy-benzo[*k*]fluoranthene as shown in the upper pathway of benzo[*k*]fluoranthene biodegradation in Fig. 3.

The benzo[*k*]fluoranthene biotransfomation product that corresponded to [M – H]^−^ = 223 was strongly detected in neutral extracts from benzo[*k*]fluoranthene-exposed cells by full scan analyses. Product ion scan analysis revealed two abundant ions at *m/z* 179 and *m/z* 151, which were indicative of losses of CO_2_ (44 Da) and CO (28 Da) from the parent deprotonated molecule. Increasing the CID energy to 20 eV did not reveal any further fragmentation even though the relative intensity of the parent deprotonated molecule was reduced to less than 5% as shown in Table 1. Figure 2B shows the mass spectrum for [M – H]^−^ = 223 and detection of the abundant diagnostic fragment ion *m/z* 151, which provided evidence for the production of an intact three-ring acenaphthylene fragmentation ion. Taken together with a proposed molecular formula of C_14_H_8_O_3_, the identity of this product was proposed to be 2-formylacenaphthylene-1-carboxylic acid. Kweon and colleagues (2007) identified 2-formylacenaphthylene-1-carboxylic acid as a PAH biotransformation product in a comprehensive investigation of *Mycobacterium vanbaalenii* strain PYR-1 metabolism of fluoranthene. Identical to as reported here, both *m/z* 179 and *m/z* 151 were reported as mass fragmentation products in that investigation.

Contrary to 2-formylacenaphthylene-1-carboxylic acid, the product that corresponded to [M – H]^−^ = 239 was only detected in acidified extracts even though it showed a molecular mass difference of only 16 Da. The results of product ion scan analyses of acidified extracts for this deprotonated molecule were similar to 2-formylacenaphthylene-1-carboxylic acid in that only two strong product ions were revealed and again one of the product ions was *m/z* 151 as shown in Fig. 2C. Considering the parent deprotonated molecule of [M – H]^−^ = 239, spectral data were indicative of consecutive losses of 2CO_2_ in that a loss of 44 Da, *m/z* 195 and a loss of 88 Da, *m/z* 151 were observed. Taking into account a molecular formula of C_14_H_8_O_4_, combined with detection of *m/z* 151, the identity of this product was proposed to be acenaphthylene-1,2-dicarboxylic acid. The presence of a carboxyl moiety at carbon position 2 of acenaphthylene-1,2-dicarboxylic acid in lieu of an aldehyde moiety as in the neutral-pH-extractable, 2-formylacenaphthylene-1-carboxylic acid, appeared to impart enough polarity to this molecule to make it difficult to extract with ethyl acetate from media that was not acidified.

Figure 2D shows the mass spectra for the unknown benzo[*k*]fluoranthene biotransformation product that corresponded to [M – H]^−^ = 229, *t_R_* = 5.6. A loss of 32 Da (*m/z* 197) from the parent deprotonated molecule indicated that [M – H]^−^ = 229 was a compound with a molar mass of 198 Da but which was detected as its methanol adduct in ESI negative ionization mode under these conditions. Abundant diagnostic fragment ions at *m/z* 185 and *m/z* 157 indicated losses of CO_2_ (44 Da) and CO_2_ plus CO (72 Da). At the same time, another abundant diagnostic fragment at *m/z* 127 was representative of the intact ring system of a naphthalene product ion following losses of all substituent groups. Considering these results, LC-MS full scan analyses of a solution of an authentic standard of 1,8-naphthalic anhydride, which has a molar mass of 198 g mol^−1^, was conducted under identical conditions in negative ionization mode. It was revealed that the predominantly detected ion was [M – H]^−^ = 229 – its methanol adduct (data not shown). After obtaining this result, product ion scan analysis of the 1,8-naphthalic anhydride authentic standard was conducted, and a diagnostic fragmentation ion pattern was observed that was identical to the pattern observed from analysis of the unknown benzo[*k*]fluoranthene product that corresponded to [M – H]^−^ = 229 (Fig. 2D and E). In addition to identical retention time data, *t_R_* = 5.6, these results allowed for identification of the product that corresponded to [M – H]^−^ = 229 in benzo[*k*]fluoranthene-exposed cell extracts as 1,8-naphthalic anhydride. Detection of 1,8-naphthalic anhydride is representative of the transformation product naphthalene-1,8-dicarboxylic acid, which is dehydrated during acidic pH solvent extraction. Finally, product ion scan analyses of the metabolite corresponding to [M – H]^−^ = 171 revealed losses of water (*m/z* 153) and CO_2_ (*m/z* 127) (Table 1). Detection of fragmentation ion *m/z* 127 provided support for the presence of an intact ring system of naphthalene as described above, and considering these results and a molecular formula of C_11_H_8_O_2_, the product that corresponded to [M – H]^−^ = 171 was proposed to be 1-naphthoic acid.

### Detection of fluoranthene and acenaphthylene biotransformation products of strain KK22

**Figure 4:**
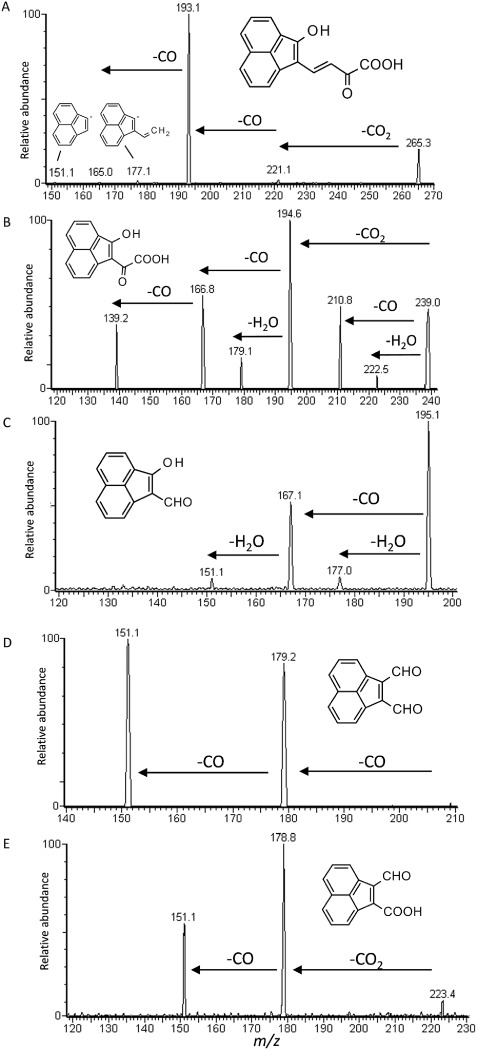
Results of LC/ESI(–)-MS/MS product ion scan analyses of extracts from strain KK22 exposure to fluoranthene. Fragmentation patterns acquired from the deprotonated molecules: (A) [M – H]^−^ = 265,; (B) [M – H]^−^ = 239; (C) [M – H]^−^ = 195; (D) [M – H]^−^ = 207 (parent deprotonated molecule is not visible); and (E) [M – H]^−^ = 223. Molecular structures proposed for each product are shown and details for all acquisitions are given in Table 2.

Investigation of the biotransformation of the structurally similar four-ring HMW PAH fluoranthene by strain KK22 revealed seven biotransformation products even though fluoranthene does not appear to support growth of strain KK22. As in the case of benzo[*k*]fluoranthene, initial dioxygenation of the exposed benzene ring of fluoranthene at the 7,8-carbon positions appeared to occur and the largest upstream product detected corresponded to [M – H]^−^ = 265. Product ion scan analysis of [M – H]^−^ = 265 revealed diagnostic fragments *m/z* 221 (loss of CO_2_), *m/z* 193 (loss of CO_2_ plus CO), *m/z* 165 (loss of CO_2_ plus 2CO) and *m/z* 151 that were indicative of losses of CO_2_, CO, aryl hydroxyl substituent and C_2_H_2_, and included evidence for a three-ring acenaphthylene fragment ion as described above in the cases of [M – H]^−^ = 223 and [M – H]^−^ = 239. Taken together with a molecular formula of C_16_H_10_O_4_, a *meta*-cleavage product of 7,8-dihydroxy-fluoranthene, 2-hydroxyacenaphthylenyl-2-oxo-but-3-enoic acid was proposed as shown in Fig. 4A.

**Table 2:** Fragmentation ions revealed by LC/ESI(–)-MS/MS product ion scan analyses of metabolites produced from the biotransformation of fluoranthene and acenaphthylene by strain KK22

Parent ion [M – H]^−^	*t_R_*^a^ (min)	CID^b^ (eV)	Diagnostic fragments from product ion scan analyses (% relative intensity)	Identity assignment
265	3.6	8	265 (M^−^, 23), 221 (M^−^ – CO_2_, 3), 193 (M^−^ – CO_2_ – CO, 100), 177 (C_14_H_9_, 2), 165 (M^−^ – CO_2_ – 2CO, 1), 151 (M^−^ – M^−^ – CO_2_ – CO – H_2_O – C_2_H_2_, 1)	2-Hydroxyacenaphthylenyl-2-oxo-but-3-enoic acid
239	3.0	20	239 (M^−^, 71), 223 (M^−^ – H_2_O, 47), 211 (M^−^ – CO, 72), 195 (M^−^ – CO_2_, 100), 179 (M^−^ – H_2_O – CO_2,_ 54), 167 (M^−^ – CO_2_ – CO, 70), 139 (M^−^ – CO_2_ – 2CO, 64)	2-Hydroxyacenaphthylenyl-2-oxo-acetic acid
229	5.6	8	229 (M^−^, 53), 215 (3), 197 (M^−^ – CH_3_OH, 13)^c^, 185 (M^−^ – CO_2_, 12), 171 (2), 157 (M^−^ – CO_2_ – CO, 100), 127 (10)	1,8-Naphthalic anhydride^d^
223	4.3	20	223 (M^−^, 12), 179 (M^−^ – CO_2_, 100), 151 (M^−^ – CO, 59)	2-Formylacenaphthylene-1-carboxylic acid^e^
207	3.5	20	207 (M^−^, < 0.1), 179 (M^−^ – CO, 90), 151 (M^−^ – 2CO, 100)	Acenaphthylene-1,2-dicarbaldehyde
195	3.4	20	195 (M^−^, 100), 177 (M^−^ – H_2_O, 8), 167 (M^−^ – CO, 52), 151 (M^−^ – CO – H_2_O, 7)	2-Hydroxyacenaphthylene-1-carbaldehyde
171	6.4	8	171 (M^−^, 100), 153 (M^−^ – H_2_O, 5), 127 (M^−^ – CO_2_, 15)	1-Naphthoic acid^d^
229^f^	5.6	8	229 (M^−^, 100), 215 (6), 197 (M^−^ – CH_3_OH, 39), 185 (M^−^ – CO_2_, 81), 171 (23), 157 (M^−^ – CO_2_ – CO, 55), 127 (6)	1,8-Naphthalic anhydride^d^
171^f^	6.2	8	171 (M^−^, 94), 153 (M^−^ – H_2_O, 1), 127 (M^−^ – CO_2_, 100)	1-Naphthoic acid^d^

a *t_R_*, retention time.

b Collision-induced dissociation energy.

c The product corresponding to [M – H]^−^ = 229 was detected as its methanol adduct.

d Products were detected from benzo[*k*]fluoranthene, fluoranthene and acenaphthylene biotransformation.

e Product was detected from benzo[*k*]fluoranthene and fluoranthene biotransformation.

f Data are for products from acenaphthylene biotransformation.

At least two other potential downstream metabolites from *meta*-cleavage of 7,8-dihydroxy-fluoranthene were detected and corresponded to the deprotonated ions [M – H]^−^ = 239 and [M – H]^−^ = 195. Six fragmentation ions were detected in the case of the fluoranthene biotransformation product that corresponded to [M – H]^−^ = 239 and indicated multiple losses of CO and a loss of CO_2_ (Fig. 4B). At the same time, separate losses of both CO_2_ (44 Da, *m/z* 195) and CO (28 Da, *m/z* 211) occurred from the parent deprotonated molecule. Considering these results and a molecular formula of C_14_H_8_O_4_, the identity of this metabolite was proposed to be the keto-acid, 2-hydroxyacenaphthylenyl-2-oxo-acetic acid, whose structure is given in Fig. 4B. The results of product ion scanning analysis of [M – H]^−^ = 195 revealed major fragments at *m/z* 177, *m/z* 167 and *m/z* 151 which indicated losses of H_2_O (−18 Da), CO (−28 Da) and the presence of an acenaphthylene fragment ion respectively from a compound with a molar mass of 208 Da. The proposed structure for this biotransformation product is shown in Fig. 4C as 2-hydroxyacenaphthylene-1-carbaldehyde, C_13_H_8_O_2_.

Evidence that *ortho*-ring cleavage of 7,8-dihydroxy-fluoranthene also occurred was provided for by the detection of two downstream biotransformation products which corresponded to the deprotonated molecules [M – H]^−^ = 207 and [M – H]^−^ = 223. Product ion scan analyses revealed that both of these deprotonated parent molecules fragmented into only two strong identical product ions each, *m/z* 151 and *m/z* 179. In the case of [M – H]^−^ = 207, consideration of a molecular formula of C_14_H_8_O_2_ combined with double losses of 28 Da from the parent deprotonated molecule (*m/z* 179 and *m/z* 151) supported an assignment of acenaphthylene-1,2-dicarbaldehyde to this fluoranthene biotransformation product. At the same time, losses of 44 Da (*m/z* 179) and 28 Da (*m/z* 151) from the parent deprotonated molecule [M – H]^−^ = 223 and a molecular formula of C_14_H_8_O_3_ indicated that this biotransformation product was 2-formylacenaphthylene-1-carboxylic acid. A similar retention time and identical mass spectrum were previously shown for the benzo[*k*]fluoranthene biotransformation product that corresponded to [M – H]^−^ = 223 (Figs. 2C and 4D and Tables 1 and 2).

Lastly, during both fluoranthene and acenaphthylene biotransformation by strain KK22, products that corresponded to the deprotonated molecules [M – H]^−^ = 229 and [M – H]^−^ = 171 were revealed. As shown in Table 2, the most abundantly detected diagnostic fragmentation ions and retention times for each pair of these products were mostly identical to each other. At the same time they were also in agreement with results obtained from product ion scan analyses of the deprotonated molecules [M – H]^−^ = 229 and [M – H]^−^ = 171 that were detected during benzo[*k*]fluoranthene biotransformation in addition to the fragmentation pattern obtained from product ion scan analysis of the authentic standard of 1,8-naphthalic acid (Tables 1 and 2). Based upon these results, these fluoranthene and acenaphthylene biotransformation products were identified as 1,8-naphthalic acid and 1-naphthoic acid and they represented further downstream points of convergence in the lower biotransformation pathway proposed for benzo[*k*]fluoranthene (Fig. 3). Accompanying data for all biotransformation products investigated from fluoranthene and acenaphthylene are given in Table 2.

### Quantitation of benzo[*k*]fluoranthene biodegradation and cell growth

A quantitative biodegradation assay was conducted whereby phenanthrene-grown strain KK22 cells were exposed to 5 mg L^−1^ benzo[*k*]fluoranthene and were monitored by whole flask extractions followed by high-performance liquid chromatography (HPLC) analyses for 10 days. Benzo[*k*]fluoranthene biodegradation occurred with approximately 10% biotransformation occurring in the first 5 days as indicated in Table 3. Between 5 and 10 days biodegradation continued with approximately 73% of benzo[*k*]fluoranthene recovered from culture media on day 10. Considering that recoveries for the abiotic controls were 90% or greater throughout the incubation period, approximately 15 to 20% of benzo[*k*]fluoranthene was biotransformed by strain KK22 under these conditions. Strain KK22 growth on benzo[*k*]fluoranthene was also investigated as described in the Experimental Procedures and results did not clearly support strain KK22 growth on benzo[*k*]fluoranthene as a sole source of carbon and energy (data not shown).

**Table 3:** Quantitative analyses of benzo[*k*]fluoranthene biodegradation by strain KK22

	Benzo[*k*]fluoranthene recovery (%)^a^		
Conditions	0 days	5 days	10 days
Benzo[*k*]fluoranthene (abiotic)	100.5 ± 2.4	90.8 ± 0.7	91.4 ± 4.6
Benzo[*k*]fluoranthene plus strain KK22	94.5 ± 2.5	77.2 ± 1.4	73.3 ± 1.6

a Average of duplicate, whole flask extractions with the recovery range indicated.

## Discussion

Initial oxidation of carbon positions 7 and 8 on the four-ring PAH fluoranthene molecule have been proposed previously for gram-positive (Kelley *et al*., 1993; López *et al*., 2005; 2006; Kweon *et al*., 2007) and gram-negative organisms (Weissenfels *et al*., 1991; Sepic *et al*., 1998) including sphingomonads (Story *et al*., 2001; van Herwijnen *et al*., 2003; Zhong *et al*., 2007; Schuler *et al*., 2008). Among the sphingomonads, *S. paucimobilis* sp. strain EPA505 biotransformed fluoranthene to form 7,8-dihydroxyfluoranthene which was followed by *meta*-ring cleavage to three-and two-ring products (Ho *et al*., 2000; Story *et al*., 2001). Cometabolic ring opening of fluoranthene by *Sphingomonas* sp. strain LB126 through 1,2-carbon position attack on the parent molecule was reported; however, ring cleavage products through 7,8-carbon position attack were not (van Herwijnen *et al*., 2003). Similar to strain EPA505, downstream products, 2-hydroxyacenaphthylene-1-carboxylic acid and naphthalene-1,8-dicarboxylic acid were reported from *Sphingomonas* sp. strain VKM B-2434 (Baboshin *et al*., 2008) and 1-acenaphthylene-1(2*H*)-one was reported from *Sphingomonas* sp. strain PheB4 (Zhong *et al*., 2007). For all of these *Sphingomonas* strains, identification of downstream metabolites indicated that 7,8-carbon position dioxygenation of fluoranthene had occurred, but with the exception of strain EPA505, few downstream flouranthene biodegradation products were identified.

*Sphingobium* species strain KK22 biotransformed benzo[*k*]fluoranthene through 8,9-carbon position initial attack on the parent molecule and this type of attack is structurally analogous to initial 7,8-carbon position attack described for fluoranthene by other gram-negative and gram-positive species. Evidence that supported this biotransformation mechanism was provided for by the identification of the downstream products 9-hydroxy-fluoranthene-8-carboxylic acid and 1,8-naphthalic anhydride for example, which may only have occurred through 8,9-carbon position initial attack on the benzo[*k*]fluoranthene molecule. The first ring fission event of the benzo[*k*]fluoranthene molecule occurred by *ortho*-ring cleavage and resulted in 8-carboxyfluoranthenyl-9-propenic acid, a product that corresponded to [M – H]^−^ = 315, and this is the first report of this product from PAH biodegradation. Production of this compound in this study is analogous to the production of the *ortho*-ring cleavage product of 1,2-dihydroxyanthracene, 3-(2-carboxyvinyl)naphthalene-2-carboxylic acid, which occurred in the first ring cleavage step of the three-ring PAH anthracene during its biotransformation by *M. vanbaalenii* strain PYR-1 (Moody *et al*., 2001). Additionally, the first ring cleavage step in the biotransformation of the three-ring PAH phenanthrene by *Arthrobacter* sp. strain P-1-1 was proposed to occur through *ortho*-ring cleavage of 1,2-and 3,4-dihydroxyphenanthrene to *ortho*-carboxyvinylnaphthoic acid metabolites (Seo *et al*., 2006). Among gram-negative species, *ortho*-ring cleavage was only proposed as the second ring cleavage event during phenanthrene biotransformation by *Burkholderia* sp. strain C3 whereby 1,2-dihydroxynaphthalene was converted into 2-carboxycinnamic acid (Seo *et al*., 2007). The proposed structure for the product that corresponded to [M – H]^−^ = 315 in this investigation is in agreement with the first ring cleavage steps proposed in the biotransformation of the HMW PAH benz[*a*]anthracene by 8,9-and/or 10,11-carbon position attack by strain KK22 (Kunihiro *et al*., 2013).

From 8-carboxyfluoranthenyl-9-propenic acid, the *o*-hydroxy-tetraaromatic acid, 9-hydroxy-fluoranthene-8-carboxylic acid, [M – H]^−^ = 261, was proposed and due to its apparent relative hydrophobicity, this *o*-hydroxy-carboxylic acid product was mostly identified in neutral extracts. In a previous study, two-and three-aromatic ring *o*-hydroxy-carboxylic acid products were mostly detected in the acidified fractions of extracts obtained during the biotransformation of benz[*a*]anthracene by this strain (Kunihiro *et al*., 2013). The greater hydrophobicity of the four-aromatic ring structure described in this investigation contributed to its lack of extractability at pH 2 and points towards a more hydrophobic character for this product which would be expected to be less mobile in the natural environment. Triaromatic *o*-hydroxy-phenanthroic and *o*-hydroxy-anthranoic acids of benz[*a*]anthracene biodegradation have only been reported as products of two *Sphingobium* species, *S. yanoikuyae* strain B1 (Mahaffey *et al*., 1988) and strain KK22 (Kunihiro *et al*., 2013), and this is the first report of 9-hydroxyfluoranthene-8-carboxylic acid production, an *o*-hydroxy-tetraaromatic acid, by a single bacterial strain.

Mass spectral data indicated that the biotransformation product(s) that corresponded to [M – H]^−^ = 265 in the biotransformation of benzo[*k*]fluoranthene were the result of *meta* cleavage of 8,9-dihydroxy-fluoranthene t hat may have occurred through decarboxylating-type hydroxylase activity (Balashova *et al*., 2001) on 9-hydroxyfluoranthene-8-carboxylic acid as shown in Fig. 3. Recent results indicate that strain KK22 may possess genes with sequence similarity to genes that code for hydroxylase enzymes of *Sphingobium* sp. strain P2 (A.H. Maeda, S. Nishi, Y. Ozeki, Y. Ohta, Y. Hatada, and R.A. Kanaly, unpubl. data); however, the enzymes in strain P2 were reported not to be involved in the conversion of *o*-polyaromatic acids (Pinyakong *et al*., 2003). From the product(s) that corresponded to [M – H]^−^ = 265, 2-formylacenaphthylene-1-carboxylic acid was formed [(M – H)^−^ = 223]. This product was detected similarly during fluoranthene biotransformation by strain KK22 and represented a point of convergence of the proposed fluoranthene pathways, with the lower pathway proposed for benzo[*k*]fluoranthene. 2-Formylacenaphthylene-1-carboxylic acid has been reported previously in the biotransformation of fluoranthene by *M. vanbaalenii* strain PYR-1 as part of a 7,8-carbon position dioxygenation and *ortho*-cleavage pathway of fluoranthene (Kweon *et al*., 2007). Similarly to Kweon and colleagues (2007), however, the predicted *ortho*-cleavage product of 7,8-dihydroxy-fluoranthene was also not detected in our investigation (Fig. 3). At the same time, the structurally similar product, 2-hydroxy-1-acenaphthoic, was reported as a product of flouranthene biodegradation from *Sphingomonas* spp. strains EPA505 and VKM B-2434 (Story *et al*., 2001; Baboshin *et al*., 2008) but was not detected during strain KK22 metabolism of benzo[*k*]fluoranthene or fluoranthene.

Oxidation of the aldehyde group of 2-formylacenaphthylene-1-carboxylic acid resulted in acenaphthylene-1,2-dicarboxylic acid, [M – H]^−^ = 239, and the production of this product during biotransformation of fluoranthene by *M. vanbaalenii* strain PYR-1 was predicted but was not recovered (Kweon *et al*., 2007). In the current study, the first documentation of this metabolite from PAH biodegradation is presented; however, it was detected as a product of benzo[*k*]fluoranthene biotransformation by strain KK22 and was not detected in fluoranthene biodegradation extracts. As shown in Fig. 3, acenaphthylene-1,2-dicarboxylic acid was likely decarboxylated to form acenaphthylene-1-carboxylic acid en route to 1,2-dihydroxy-acenaphthyelene, which was predicted to have occurred based upon the confirmation of 1,8-naphthalic anhydride in the culture media extracts. 1,8-Naphthalic anhydride was detected in extracts from all three PAHs investigated and represented a key point of downstream pathway convergence whereby its detection during fluoranthene and acenaphthylene biotransformation confirmed the lower pathway proposed for benzo[*k*]fluoranthene. Naphthalene-1,8-dicarboxylic acid detected directly or as 1,8-naphthalic anhydride as products of both fluoranthene and acenaphthylene biotransformation have been reported previously, but not for benzo[*k*]fluoranthene (Story *et al*., 2001; López *et al*., 2006; Poonthrigpun *et al*., 2006; Kweon *et al*., 2007; Lee *et al*., 2007). The identification of 1,8-naphthalic anhydride in benzo[*k*]fluoranthene-exposed cell extracts in this investigation provided further support for benzo[*k*]fluoranthene biotransformation to downstream three-ring and two-ring products by strain KK22, and its detection was indicative of naphthalene-1,8-dicarboxylic acid production from acenaphthoquinone as shown in Fig. 3. First evidence of acenaphthoquinone production from acenaphthylene by *S. yanoikuyae* strain B1 was reported in 1984 by Schocken and Gibson. Finally, 1-naphthoic acid was also detected in extracts from benzo[*k*]fluoranthene, fluoranthene and acenaphthylene biodegradation, and further supported the pathway proposed for benzo[*k*]fluoranthene biotransformation by strain KK22.

Fluoranthene and acenaphthylene biotransformation by strain KK22 were examined independently in this study, and results provided support for the proposed benzo[*k*]fluoranthene lower biotransformation pathway and provided further insight into the degradation mechanisms of PAHs by this strain. Based upon the identification of downstream products, fluoranthene biotransformation most likely occurred through 7,8-carbon position dioxygenation, leading to the formation of *cis*-7,8-fluoranthene-dihydrodiol and 7,8-dihydroxyfluoranthene, which underwent both *ortho*-and *meta*-ring cleavage. A dicarboxylic acid *ortho*-ring cleavage product was not directly detected in culture extracts; however, evidence for *ortho*-ring cleavage of 7,8-dihydroxy-fluoranthene was provided for by the identification of the two downstream three-ring products that corresponded to [M – H]^−^ = 207 and [M – H]^−^ = 223, acenaphthylene-1,2-dicarbaldehyde and 2-formylacenaphthylene-1-carboxylic acid respectively. Oxidation of acenaphthylene-1,2-dicarbaldehyde resulted in 2-formylacenaphthylene-1-carboxylic acid but may have also resulted in the production of 2-hydroxyacenaphthylene-1-carbaldehyde, [M – H]^−^ = 195, en route to acenaphthoquinione, and this branch of the pathway is indicated in Fig. 3. 2-Formylacenaphthylene-1-carboxylic acid was also identified as a product of fluoranthene biodegradation in a previous study (Kweon *et al*., 2007).

A *meta*-cleavage product of 7,8-dihydroxy-fluoranthene corresponding to [M – H]^−^ = 265, 2-hydroxyacenaphthylenyl-2-oxo-but-3-enoic acid was identified in culture extracts from strain KK22. Detection of the *m/z* 151 product ion during mass fragmentation combined with the lack of evidence of downstream products from a 2,3-carbon position ring-cleavage event supported this assignment. 2-Hydroxyacenaphthylenyl-2-oxo-but-3-enoic acid was predicted to have occurred during biodegradation of fluoranthene by *S. paucimobilis* strain EPA505 but was not detected (Story *et al*., 2001). Kweon and colleagues (2007) later confirmed this metabolite from fluoranthene biotransformation by *M. vanbaalenii* strain PYR-1. Two potential *meta*-(or *ortho-*)ring cleavage downstream products, 2-hydroxyacenaphthylenyl-2-oxo-acetic acid [M – H]^−^ = 239 and 2-hydroxyacenaphthylene-1-carbaldehyde, [M – H]^−^ = 195 were detected in the current study and represented new metabolites in the biodegradation of fluoranthene by bacteria. In Fig. 3, they are shown as *meta*-ring cleavage metabolites of 7,8-dihydroxy-fluoranthene; however, they may also occur via the *ortho*-ring cleavage pathway and not necessarily through acenaphthylene-1,2-dicarbaldehyde as shown.

Finally, two diarene downstream products that were characterized in previous studies through dioxygenation of acenaphthylene (Poonthrigpun *et al*., 2006) or through production of acenaphthoquinone (López *et al*., 2006; Poonthrigpun *et al*., 2006; Kweon *et al*., 2007) were also detected in this study. Similarly to benzo[*k*]fluoranthene biotransformation by strain KK22, the deprotonated molecules [M – H]^−^ = 229 and [M – H]^−^ = 171 were identified from both fluoranthene and acenaphthylene biotransformations as 1,8-naphthalic anhydride and 1-naphthoic acid, respectively, and these results provided further evidence for the metabolic capability of strain KK22 in regard to the lower pathway proposed for benzo[*k*]fluoranthene biotransformation.

In conclusion, the bacterial isolate *Sphingobium* species strain KK22 biotransformed the five-ring HMW PAH benzo[*k*]fluoranthene, and LC/ESI(–)-MS(/MS) was shown to be a useful tool to investigate the biotransformation products that were produced from it and from the structurally related PAHs fluoranthene and acenaphthylene to construct a pathway for benzo[*k*]fluoranthene biotransformation. Qualitative investigations that utilized tandem mass spectrometry showed that strain KK22 was capable of producing four-, three-and two-ring biotransformation products from benzo[*k*]fluoranthene and quantitative analyses confirmed benzo[*k*]fluoranthene biodegradation. The results from qualitative analyses provided evidence that the first and second aromatic-ring cleavage events of benzo[*k*]fluoranthene biotransformation occurred by *ortho* and *meta* mechanisms, respectively, ultimately leading to three-ring products including the previously unreported, acenaphthylene-1,2-dicarboxylic acid. Through decarboxylation and subsequent oxidation steps, acenaphthoquinone by way of 1,2-dihydroxy-acenaphthylene was produced, and this led to spontaneous ring-opening and production of the two-ring products, 1,8-naphthalic acid, naphthalene-1,8-dicarboxylic acid and 1-naphthoic acid. For comparative purposes, biotransformation pathways for fluoranthene and acenaphthylene by strain KK22 were also elucidated by LC/ESI(–)-MS(/MS). Convergence of the pathways of these PAHs with the pathway proposed for benzo[*k*]fluoranthene biotransformation was revealed, and these data confirmed the lower pathway proposed for benzo[*k*]fluoranthene by strain KK22 in this study. Cell growth of strain KK22 on benzo[*k*]fluoranthene was not demonstrated even though downstream metabolites such as naphthalene-1,8-dicarboxylic acid were detected, and it was concluded that the rate-limiting step in benzo[*k*]fluoranthene biotransformation occurred early, most likely with the production of 9-hydroxy-fluoranthene-8-carboxylic acid. This product comprised both hydrophobic and hydrophilic groups but overall appeared to be relatively hydrophobic compared with its 3-and 2-aromatic ring *o*-hydroxy-carboxylic acid counterparts produced from other PAHs during biodegradation. The results of these investigations contribute to our knowledge of the pathways of HMW PAH biodegradation by bacteria.

## Experimental procedures

### Chemicals, growth media and bacterial strain

Benzo[*k*]fluoranthene (≥ 98.5% purity), fluoranthene (≥ 97% purity), ethyl acetate and methanol (HPLC grade or higher) were purchased from Wako Chemical (Osaka, Japan). Acenaphthylene (≥ 98% purity) was from Tokyo Chemical Industries (Tokyo, Japan), and phenanthrene (97% purity) was purchased from Sigma-Aldrich (St Louis, MO, USA). Strain KK22 was originally isolated from a soil bacterial consortium that grew on diesel fuel and biodegraded PAHs (Kanaly *et al*., 1997; 2000; Kanaly and Watanabe, 2004), and details in regard to strain KK22 are given in Kunihiro and colleagues (2013). Strain KK22 was maintained on phenanthrene as the sole source of carbon and energy, 300 mg l^−1^, in Stanier's basal medium (SBM; Atlas, 1993) by continuous rotary shaking at 150 r.p.m. at 30°C in the dark. To prepare cells for biodegradation assays, phenanthrene dissolved in diethyl ether was applied to the bottoms of sterilized Erlenmeyer flasks via microsyringe (Hamilton, Reno, NV, USA) under aseptic conditions. Diethyl ether was evaporated via filter-sterilized nitrogen gas and followed by addition of SBM and inoculum.

### Monitoring of indicators of cell growth

Absorbance monitoring and total protein monitoring were conducted in triplicate under conditions identical to those described for qualitative benzo[*k*]fluoranthene biotransformation assays. To monitor absorbance, culture supernatant fluids in 600 μl aliquots were aseptically sampled and analysed by UV-visible spectrophotometry using an optical density equal to 620 nm on a V-530 UV/VIS spectrophotometer (Jasco, Tokyo, Japan) through for 4 weeks. Total protein was also monitored through 3 weeks by the bicinchoninic method according to the manufacturer's instructions (Sigma-Aldrich).

### Analyses of benzo[*k*]fluoranthene biotransformation products

Strain KK22 was grown on 250 mg l^−1^ phenanthrene in 2 l flasks that were prepared as described above for 6 days. After incubation, cells were aseptically filtered through glass wool, harvested by centrifugation (8700 × *g*, 10 min, 4°C), resuspended in 30 ml of phosphate buffer (50 mM, pH 7) and followed by three cell washing and centrifugation steps at 5700 × *g*, 4°C for 10 min, 8 min and 8 min each. After the final washing step, cells were resuspended in SBM and incubated by rotary shaking for approximately 16 h at 30°C and 175 r.p.m. to deplete the media of remaining metabolites. Biotransformation was monitored in cultures that consisted of 2 l size Erlenmeyer flasks that each contained 250 ml of SBM each plus strain KK22 cells and 20 mg l^−1^ of benzo[*k*]fluoranthene in *N*,*N*-dimethylformamide, (< 0.1% *v/v*; Wako, Osaka, Japan), depending upon treatment conditions as described as follows. Cultures were prepared either with strain KK22 cells plus benzo[*k*]fluoranthene or strain KK22 cells without benzo[*k*]fluoranthene (biotic controls), whereby the absorbance at OD_620_ of harvested and washed cells was adjusted to approximately 0.10 by analysis of cell suspensions using a V-530 model UV-visible spectrophotometer (Jasco). Abiotic controls consisted of benzo[*k*]fluoranthene without cells. All cultures were incubated by rotary shaking at 30°C at 175 r.p.m. in the dark and were sampled multiple times over a period of 4 weeks, whereby 20 ml of culture supernatant liquid were aseptically removed at each sampling time and extracted at neutral pH with 40 ml of ethyl acetate. Organic and aqueous phases were separated in glass separatory funnels; the organic phases were passed through anhydrous sodium sulphate that was prepared by overnight drying at 50°C, and the aqueous phases were extracted a second time identically as before. Extracts were pooled, concentrated *en vacuo* via rotary evaporation (Eyela, Tokyo, Japan), residues were resuspended in methanol and passed through 0.45 μm PTFE syringe filters (Whatman, Maidstone, UK) into brown glass vials. To recover polar metabolites, culture fluids were re-extracted in an identical manner following acidification of the extracted culture media to pH 2 with concentrated hydrochloric acid.

Neutral and acidified sample extracts were analysed separately by LC/ESI-MS using a Waters 2690 Separations Module delivery system in line with a Waters 2998 photo diode array detector (Waters Corp., Milford, MA, USA) that was interfaced with a Quattro Ultima triple stage quadrupole mass spectrometer (Micromass, Manchester, UK). Extracts were eluted isocratically in 77% methanol/water and separated by using an XSelect CSH C18 4.6 × 150 column (Waters) that was in line with a Security Guard Cartridge System pre-column fitted with a widepore C18 cartridge (Phenomenex, Torrance, CA, USA). Full scan analyses were conducted over a range of 50–500 *m/z* in negative electrospray ionization mode. Nitrogen was used as the nebulizing gas; the ion source temperature was 130°C, the desolvation temperature was 350°C and the cone voltage was operated at 40 V. Nitrogen gas was also used as the desolvation gas (600 l h^−1^) and the cone gas (60 l h^−1^). Results from full scan analyses were examined to determine putative mass ions of interest by comparing the results of analyses of extracts from cells exposed to benzo[*k*]fluoranthene with the results of analyses of extracts from unexposed cells and abiotic controls. Through this process, multiple putative ions of interest were identified and targeted for tandem mass analyses.

LC/ESI-tandem mass product ion and precursor ion scan analyses were conducted in negative ionization mode [LC/ESI(–)-MS/MS] by using CID under mass conditions similar to as described above. Argon gas was used as the collision cell gas, and varying collision cell energies were employed generally over a range of 5–20 eV depending upon the sample. Mass spectral fragmentation patterns from product ion and precursor ion scanning at varying collision energies were analysed to aid in the determination of the molecular structures of unknown biotransformation products.

### Analyses of fluoranthene and acenaphthylene biotransformation products and identification of downstream products with authentic standards

Strain KK22 biotransformation assays of fluoranthene and acenaphthylene were prepared similarly to those prepared for benzo[*k*]fluoranthene, except that the concentrations of each were 250 mg l^−1^ and 50 mg l^−1^, respectively, and cultures were incubated in 30 ml of SBM in 300 ml size Erlenmeyer flasks for 72 h. Samples were taken at 24, 48 and 72 h, extracted and analysed by LC/ESI(–)-MS(/MS) as described above. The elution times of fluoranthene and acenaphthylene were approximately 36 and 7 min respectively.

To confirm the identity of 1,8-naphthalic anhydride in sample extracts of PAH biotransformation, an authentic standard (Kanto Chemical, Tokyo, Japan, respectively) was prepared in methanol at a concentration of 1 mg l^−1^ and analysed by LC/ESI(–)-MS(/MS) under full scan and product ion scan mass conditions as described above.

### Quantitation of benzo[*k*]fluoranthene biodegradation

Quantitation of benzo[*k*]fluoranthene biodegradation was investigated in duplicate flasks that were prepared in 100 ml size Erlenmeyer flasks that each contained 20 ml of SBM each plus 5 mg l^−1^ benzo[*k*]fluoranthene as described above. Treatments that consisted of benzo[*k*]fluoranthene without cells served as abiotic controls. All cultures were incubated by rotary shaking at 30°C at 175 r.p.m. in the dark, and whole flask extractions were conducted at 0, 5 and 10 days. Pyrene in ethyl acetate was added as an extraction standard, followed by addition of 20 ml of ethyl acetate to each culture (1:1). Flasks were shaken overnight at 20°C and 150 r.p.m. in the dark. Organic and aqueous phases were separated, and extracts were prepared as described above.

Quantitative analyses were performed by HPLC with UV detection using a Waters 2690 Separations Module delivery system in line with a Shimadzu SPD-10A UV-VIS detector (Shimadzu Corp., Kyoto, Japan). Extracts were eluted isocratically in 77% methanol/water and separated on a Crestpak C18S 4.6 × 150 mm column (Jasco). The flow rate was 0.3 ml min^−1^, each sample run was 110 min and the retention times of benzo[*k*]fluoranthene and pyrene were approximately 91 and 39 min, respectively, under these conditions.

## Conflict of interest

None declared.
